# Supplementary material for: DMRforPairs: identifying Differentially Methylated Regions between unique samples using array based methylation profiles
Source: BMC Bioinformatics. 2014 May 15;15:141. doi: 10.1186/1471-2105-15-141 (PMC4046028; doi:10.1186/1471-2105-15-141)
Supplement: Additional file 2 — DMRforPairs output for the comparison of A431-MCF7 and NA17018-NA17105. Please start from the HTML files in each folder. Available via the BMC Bioinformatics website. [file 1471-2105-15-141-S2.zip › 1394847754114233_MOESM2_ESM/A431_MCF7/figures/19477.pdf]

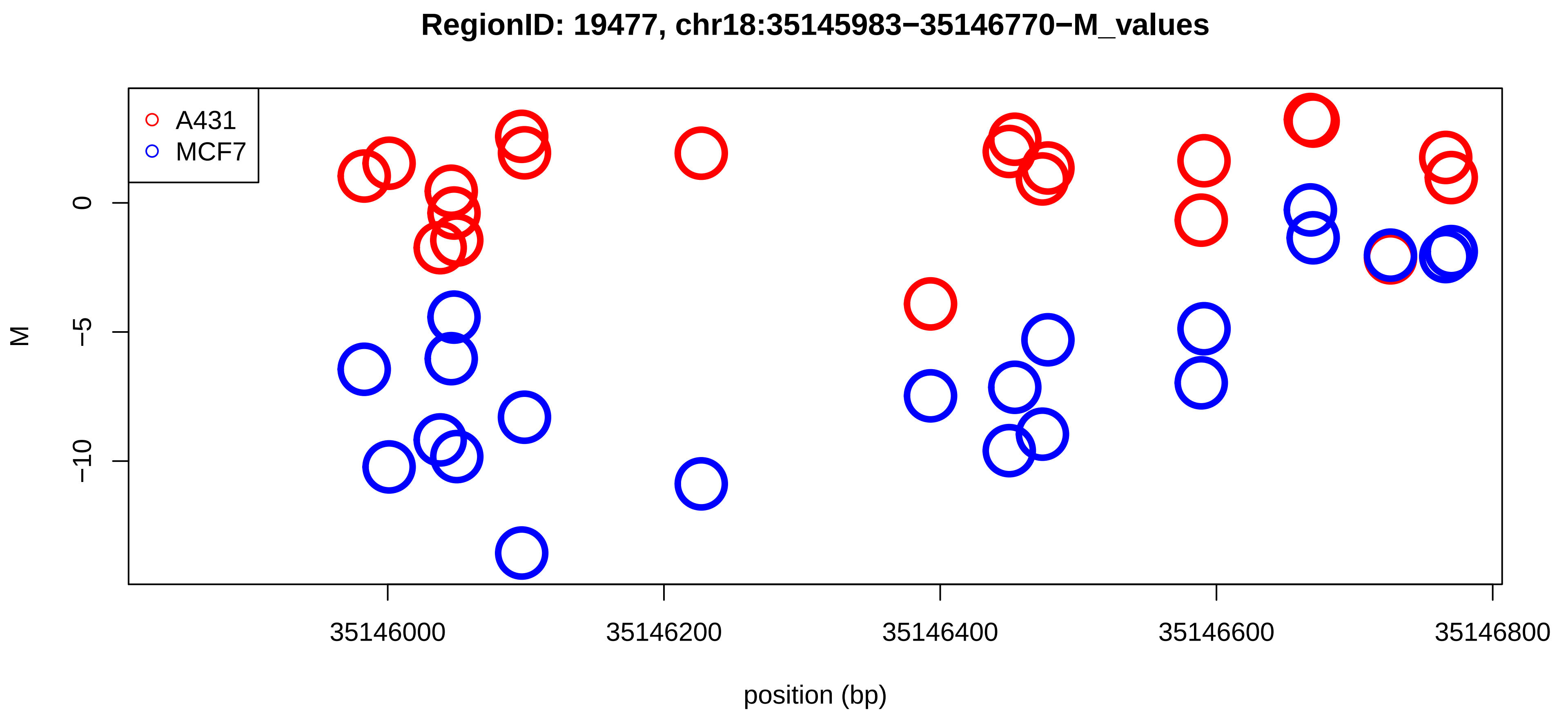
RegionID: 19477, chr18:35145983−35146770−M_values
A431
MCF7
M
0
−5
−10
35146000
35146200
35146400
35146600
35146800
position (bp)

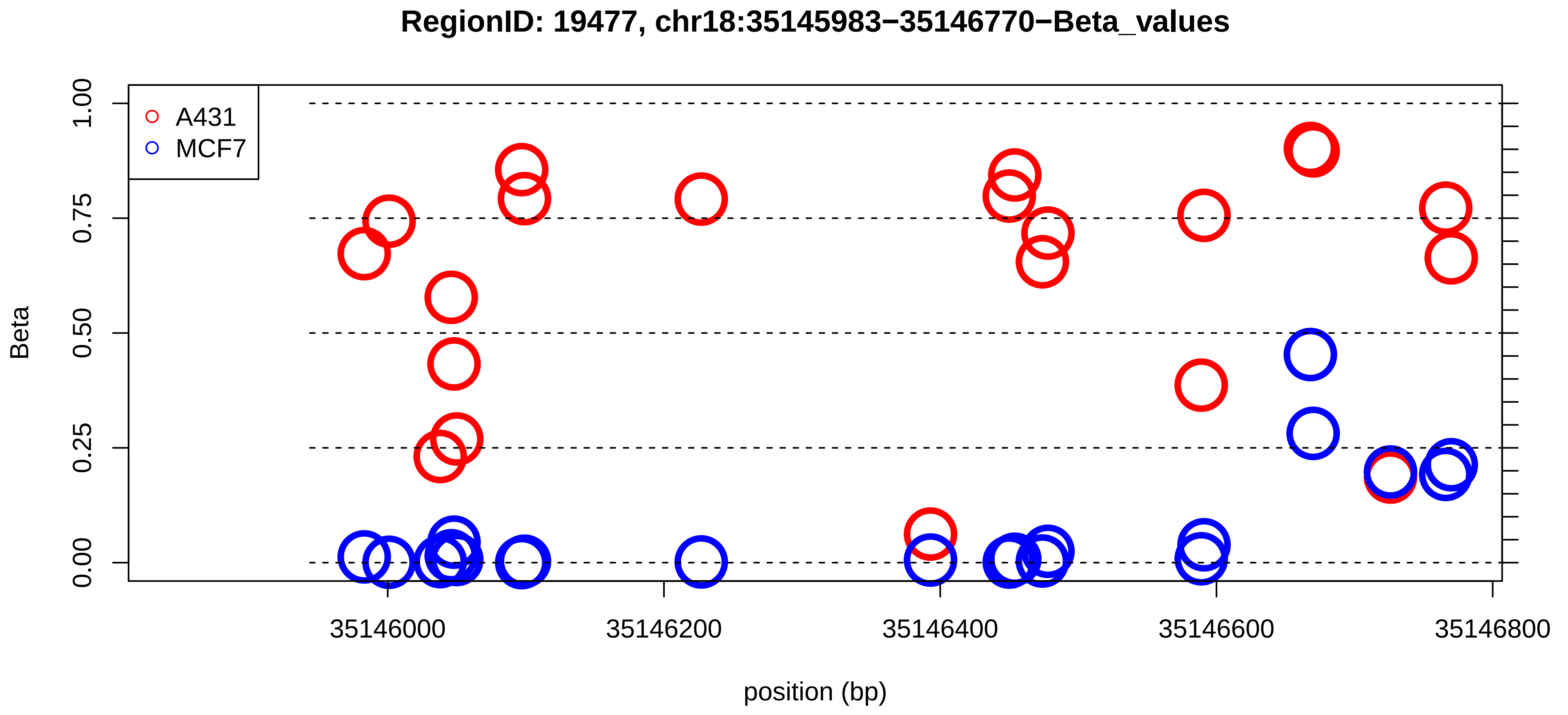
RegionID: 19477, chr18:35145983−35146770−Beta_values
A431
MCF7
Beta
0.00
0.25
0.50
0.75
1.00
35146000
35146200
35146400
35146600
35146800
position (bp)

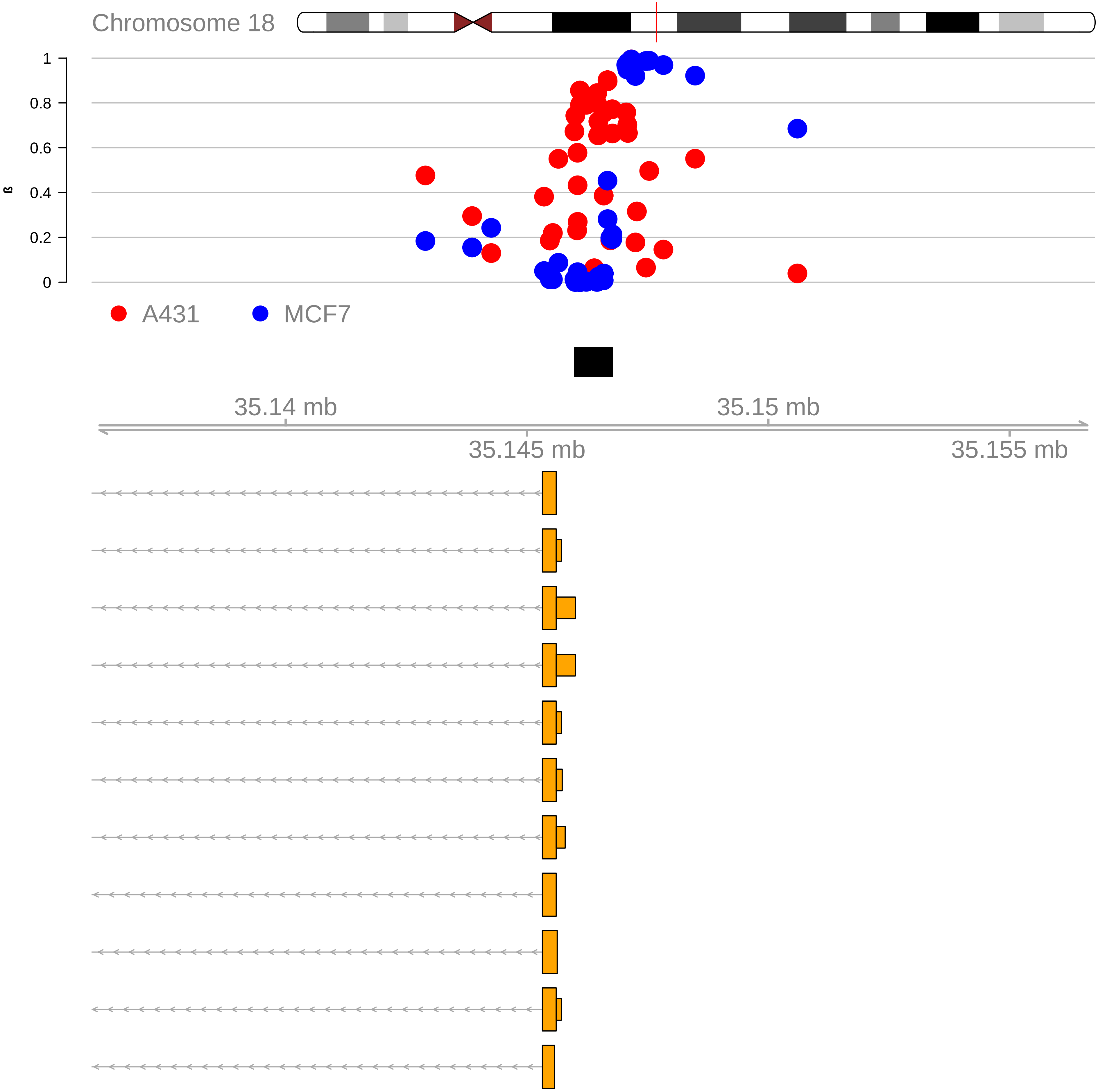
Chromosome 18
1
0.8
0.6
0.4
0.2
0
β
A431
MCF7
35.14 mb
35.15 mb
35.145 mb
35.155 mb
